# Inhibition of Zika virus by *Wolbachia* in *Aedes aegypti*

**DOI:** 10.15698/mic2016.07.513

**Published:** 2016-06-27

**Authors:** Eric Pearce Caragata, Heverton Leandro Carneiro Dutra, Luciano Andrade Moreira

**Affiliations:** 1 Mosquitos Vetores: Endossimbiontes e Interação Patógeno-Vetor, Centro de Pesquisas René Rachou – Fiocruz, Belo Horizonte, MG, Brazil.

**Keywords:** Zika virus, mosquito-transmitted disease, Aedes aegypti, Wolbachia

## Abstract

Through association with cases of microcephaly in 2015, Zika virus (ZIKV) has transitioned from a relatively unknown mosquito-transmitted pathogen to a global health emergency, emphasizing the need to improve existing mosquito control programs to prevent future disease outbreaks. The response to Zika must involve a paradigm shift from traditional to novel methods of mosquito control, and according to the World Health Organization should incorporate the release of mosquitoes infected with the bacterial endosymbiont *Wolbachia* *pipientis*. In our recent paper [Dutra, HLC *et al.*, Cell Host & Microbe 2016] we investigated the potential of *Wolbachia* infections in *Aedes aegypti* to restrict infection and transmission of Zika virus recently isolated in Brazil. *Wolbachia* is now well known for its ability to block or reduce infection with a variety of pathogens in different mosquito species including the dengue (DENV), yellow fever, and chikungunya viruses, and malaria-causing *Plasmodium*, and consequently has great potential to control mosquito-transmitted diseases across the globe. Our results demonstrated that the *w*Mel *Wolbachia* strain in Brazilian *Ae. aegypti* is a strong inhibitor of ZIKV infection, and furthermore appears to prevent transmission of infectious viral particles in mosquito saliva, which highlights the bacterium’s suitability for more widespread use in Zika control.

## *Wolbachia* can limit vector-borne disease transmission

*Aedes aegypti* mosquitoes infected with the *w*Mel *Wolbachia* strain are already present in the field in several countries as part of a mosquito control strategy designed to reduce the high disease burden of dengue (www.eliminatedengue.com). *w*Mel is an ideal agent for mosquito control as it can rapidly and stably spread into wild mosquito populations through cytoplasmic incompatibility, has little impact on host competitiveness, and offers a high degree of inhibition of DENV. In Brazil, these mosquitoes have been released in two suburbs of Rio de Janeiro since late 2014, as part of the initial characterization of the suitability of the bacterium for use in Latin American mosquito populations. Given the recent emergence of Zika in the region, it was important to understand whether *w*Mel caused a similar inhibition of ZIKV infections in Brazilian mosquitoes.

## ZIKV infection in mosquito tissues

We conducted experimental oral infections using two currently circulating ZIKV isolates (BRPE and SPH) that were isolated from patient blood during the late 2015 outbreak. These viruses were maintained in mosquito cells for a short period of time, and fresh, infected supernatant was mixed directly with human blood prior to mosquito feeding. Our use of recently isolated, unfrozen virus meant that our experimental infection process approximated natural virus acquisition for mosquitoes in the field. Likewise, both the *Wolbachia*-infected (*w*Mel_Br) and -uninfected (Br) mosquito lines that we used had a genetic background that was representative of mosquitoes near the *Wolbachia* release sites in Rio de Janeiro. The Br line was derived from eggs collected in this location in early 2016, and genetic similarity with the *w*Mel_Br line was maintained by regular introduction of Br males into *w*Mel_Br cages. Consequently, our experiments provided insight into how *Wolbachia*-infected mosquitoes might respond to ZIKV infection in nature.

**Figure 1:**
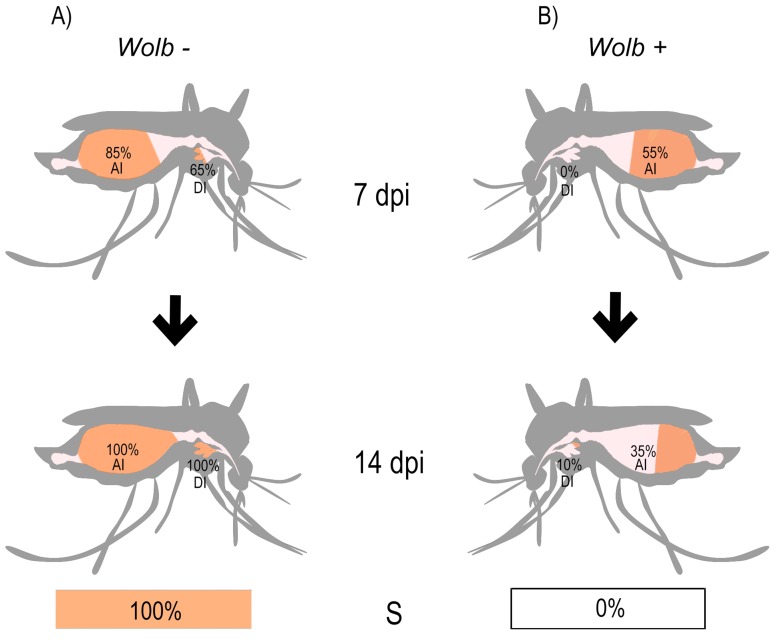
FIGURE 1: Schematic depicting the prevalence of Zika virus infection in *Wolbachia*-uninfected (A, *Wolb -*) and *Wolbachia*-infected (B, *Wolb *+) *Aedes aegypti*. Data for the BRPE ZIKV isolate were obtained through RT-qPCR analysis. Percentage values represent abdominal infection rates (AI), and disseminated infection rates (DI) at 7 and 14 days post-infection (dpi), or rate of subsequent infection after saliva was injected into naïve *Wolbachia*-uninfected mosquitoes at 14 dpi (S).

We used a Taqman-based RT-qPCR assay to quantify ZIKV levels in mosquito abdomens and heads/thoraces, at 7 and 14 days post-infection, with the latter tissues providing a representation of disseminated infection (Fig. 1). Our data indicated that *w*Mel infection decreased the rate of ZIKV abdominal infection for both isolates, at both time points, and interestingly ZIKV infection rates in *w*Mel_Br mosquitoes remained low at 14 dpi, highlighting the ability of *Wolbachia* to hinder ZIKV establishment in mosquito tissues.

Similarly, our data showed very low prevalence (proportion of mosquitoes infected) of disseminated ZIKV infection in *w*Mel_Br mosquitoes at 7 dpi (BRPE - 0%, SPH - 5%), and encouragingly this only increased slightly by 14 dpi (BRPE - 10%, SPH - 25%). By comparison, disseminated infection levels were high amongst Br mosquitoes at both time points, reinforcing the concept of a rapid ZIKV extrinsic incubation period (EIP - the time between virus intake and when the mosquito can transmit it to new hosts) in *Ae. aegypti*. Our data suggest that *w*Mel is effective at preventing ZIKV from exiting the midgut, and further experimentation may reveal that the bacterium actually prolongs the EIP, which would greatly reduce the chance of viral transmission.

Looking at ZIKV titers, we saw that Br mosquitoes experienced severe infection, with median infection levels on the order of 10^11^ viral genomes per tissue. Those few *w*Mel_Br mosquitoes that did end up infected experienced substantially reduced ZIKV titers; a comparative decrease of 5-6 log copies per tissue. This difference highlights the reduction in viral burden associated with *w*Mel, and we speculate that this could potentially occur because the bacterium limits the ability of ZIKV to replicate and invade cells, as has previously been hypothesized for other viruses in mosquitoes.

## ZIKV transmission

While tissue infection data are an important gauge for the effectiveness of inhibition by *Wolbachia*, the effect of *w*Mel on viral transmission is a more epidemiologically important factor, and one that we examined using two different methods. First, we analyzed BRPE ZIKV isolate titers directly in mosquito saliva samples obtained for *w*Mel_Br and Br mosquitoes. We saw that although the virus was detected in approximately half of the *w*Mel saliva samples, *w*Mel significantly reduced ZIKV prevalence and intensity of infection in saliva.

We then injected some of these saliva samples into ZIKV-uninfected Br mosquitoes to see if the saliva contained infectious virus - similar to a plaque assay but offering the benefit of demonstrating viral replication *in vivo* rather than *in cellula*. We saw that all of Br mosquito saliva contained infectious virus, while no *w*Mel_Br saliva produced subsequent infections. This indicated that *w*Mel_Br mosquito saliva did not contain infectious viral particles, and that the virus we had detected in the other *w*Mel_Br saliva was likely non-infectious. Other studies have demonstrated that *w*Mel-infected mosquitoes have lower rates of expectoration, and consequently have a reduced ability to transmit DENV, which may be the case here as well. The absence of infectious virus in *w*Mel_Br saliva was perhaps the most important finding in our paper, and is a clear indicator of the potential of *Wolbachia* to limit ZIKV transmission in nature.

## Conclusions and perspectives

Our results suggest that there is an opportunity to utilize the infrastructure for releasing *Wolbachia*-infected mosquitoes for dengue control purposes that is already present in Brazil and other countries, and expand releases to target Zika. The endpoint of this strategy would be the replacement of wild mosquito populations in areas with high levels of ZIKV transmission with *w*Mel-infected, ZIKV-resistant mosquitoes. This greater prevalence of *w*Mel-infected *Ae. aegypti* would also beneficially serve to reduce the transmission of dengue and chikungunya, both of which remain an ongoing concern in Latin America.

It should be noted that our study took place under controlled laboratory conditions, and only used two ZIKV isolates. To gain greater confidence in the inhibitory ability of *w*Mel we need to understand the plasticity of ZIKV inhibition when *w*Mel-infected mosquitoes encounter the virus naturally, under different environmental conditions, and against a broader range of viral genotypes. Analysis of the ability of *w*Mel to block ZIKV transmission under all of these conditions will go a long way towards determining what level of reduction in ZIKV transmission might be obtained if *Wolbachia*-infected mosquitoes were deployed across large areas.

It is important to note that *Wolbachia*-based mosquito control has not been implemented on a large scale in Latin America, and an expansion in the associated rearing, distribution and surveillance processes will require careful planning prior to deployment. This technique is likely something that will work best in combination with existing or novel mosquito control strategies, and to that end it may be possible to identify areas of overlap, where roll-out costs might be reduced. These alternative strategies include insecticides, the release of RIDL mosquitoes, and the incompatible insect technique, all of which will likely be used to control mosquito populations in the face of incipient outbreaks. *Wolbachia*-based population replacement is perhaps better suited for use as a long-term preventative measure, given that it typically takes several months for *Wolbachia* to become established and self-sustaining in mosquito populations. It is this unique characteristic that will likely see *Wolbachia* become an essential part of future integrated mosquito control programs.

